# An adult case of Bland–White–Garland syndrome with Vieussens’ arterial ring

**DOI:** 10.1093/ehjcr/ytae468

**Published:** 2024-09-02

**Authors:** Hikaru Hagiwara, Hirokazu Komoriyama, Yoshiya Kato, Toshihisa Anzai

**Affiliations:** Department of Cardiovascular Medicine, Kushiro City General Hospital, 1-12, Shunkodai, Kushiro 085-0822, Japan; Department of Cardiovascular Medicine, Faculty of Medicine and Graduate School of Medicine, Hokkaido University, Kita-15, Nishi-7, Kita-ku, Sapporo 060-8638, Japan; Department of Cardiovascular Medicine, Kushiro City General Hospital, 1-12, Shunkodai, Kushiro 085-0822, Japan; Department of Cardiovascular Medicine, Kushiro City General Hospital, 1-12, Shunkodai, Kushiro 085-0822, Japan; Department of Cardiovascular Medicine, Faculty of Medicine and Graduate School of Medicine, Hokkaido University, Kita-15, Nishi-7, Kita-ku, Sapporo 060-8638, Japan; Department of Cardiovascular Medicine, Faculty of Medicine and Graduate School of Medicine, Hokkaido University, Kita-15, Nishi-7, Kita-ku, Sapporo 060-8638, Japan

## Case description

Bland–White–Garland (BWG) syndrome is a rare congenital cardiac anomaly for which only a few patients survive into adulthood.^1^ This report presents the rare case of a 58-year-old patient with BWG syndrome who did not receive surgical treatment for the condition.

**Figure 1:**
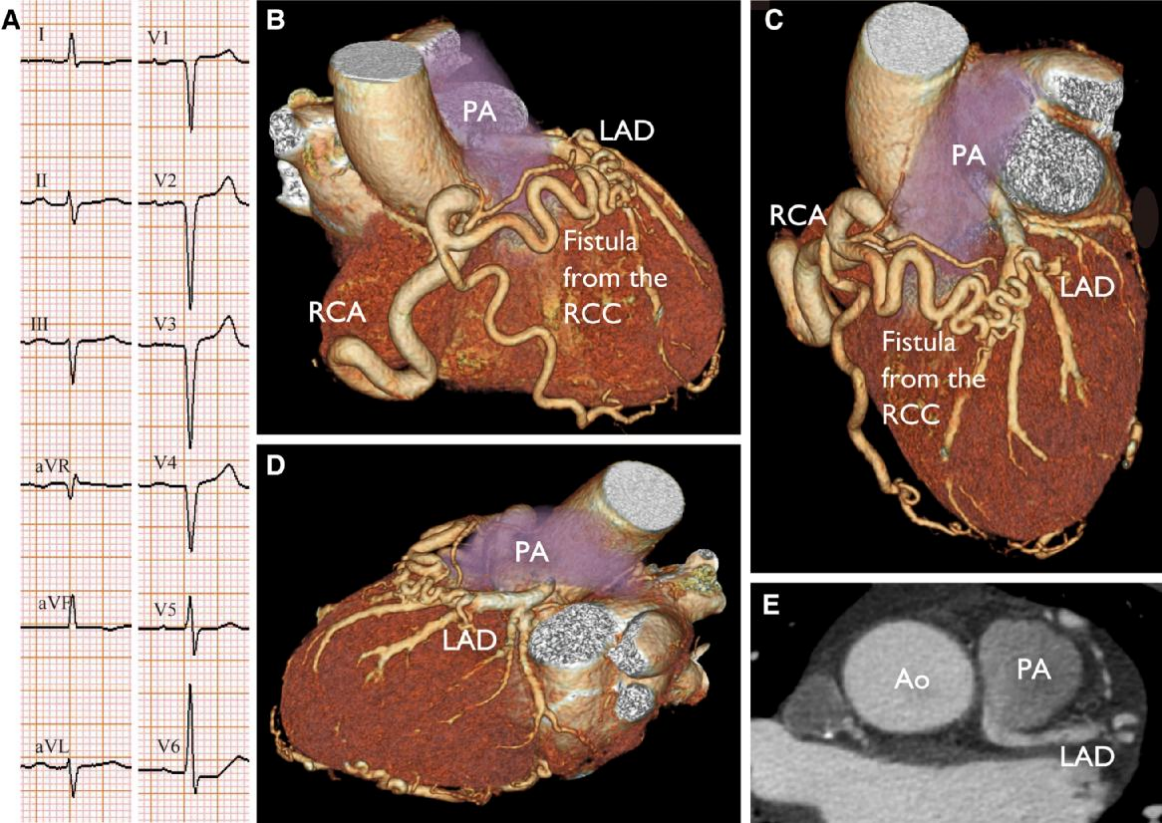
Twelve-lead electrocardiography (*A*) and volume-rendered computed tomography of the heart of a 58-year-old patient with Bland–White–Garland syndrome showing complex anomalous vessels. The conus artery arises from the right sinus of Valsalva and connects to the proximal portion of the left anterior descending coronary artery (*B, C*). The left coronary artery anomalously originates from the pulmonary artery (*D, E*). Ao, aorta; RCA, right coronary artery; RCC, right coronary cusp; LAD, left anterior descending artery; PA, pulmonary artery.

A 58-year-old woman visited a local clinic complaining of shoulder pain and found to have poor R-wave progression electrocardiogram (*Figure 1A*) and was referred to our hospital. Echocardiogram showed anterior wall motion abnormality and decreased left ventricular ejection fraction of 42%. Coronary angiography revealed right coronary artery dominance (see Supplementary material online, *Video S1*) and no visible left main stem artery (see Supplementary material online, *Video S2*). Furthermore, a connection was observed between the conus branch and the left coronary and pulmonary arteries (see Supplementary material online, *Videos S3* and *S4*). Volume-rendered computed tomography showed a Vieussens’ arterial ring (*Figure 1B* and *C*), a connection between the conus artery and the diagonal branches of the left anterior descending coronary artery, and an anomalous origin of the left coronary artery from the pulmonary artery (*Figure 1D* and *E*).

Patients with BWG syndrome can die abruptly; ∼90% of them die at an average age of 35 years.^1^ The present case had rich collateral circulation from the conus artery, which increased blood flow in the left coronary artery and reduced the degree of myocardial ischaemia, enabling her to live up to 58 years of age. Previous reports have suggested that patients with BWG syndrome should undergo surgical intervention, regardless of the presence or absence of clinical symptoms or complications, to prevent the development of heart failure and reduce the risk of sudden cardiac death; therefore, surgical treatment was planned in this case.^1^

## Supplementary Material

ytae468_Supplementary_Data

## Data Availability

Data sharing is not applicable as no data sets were generated or analysed for this case report.

## Supplementary material

**Consent:** Informed consent was provided by the patient.

**Funding:** None declared.
